# Supplementary material for: Sentinel node approach to monitoring online COVID-19 misinformation
Source: Sci Rep. 2022 Jun 14;12:9832. doi: 10.1038/s41598-022-12450-8 (PMC9194351; doi:10.1038/s41598-022-12450-8)
Supplement: Supplementary file 1 — Supplementary Information 1. [file 41598_2022_12450_MOESM1_ESM.pdf]

Supplementary Materials: Sentinel node approach to monitoring online COVID-19 misinformation

Matthew T. Osborne, Samuel S. Malloy,
Erik C. Nisbet, Robert M. Bond, Joseph H. Tien

March 16, 2022

1 Appendix/Supplementary Material

1.1 Linked domain score

1.1.1 PCA Tables

The domains corresponding to the 30 most positive and negative entries of the first PCA direction from the linked domain PCA analysis are given in Tables 1 and 2.

Domain	First PCA Value
msnbc.com	−0.0355
vote.org	−0.0300
rollcall.com	−0.0295
vogue.com	−0.0295
actblue.com	−0.0294
buzzfeednews.com	−0.0284
salon.com	−0.0275
slate.com	−0.0275
esquire.com	−0.0274
essence.com	−0.0273
newyorker.com	−0.0267
thedailybeast.com	−0.0263
Vote.org	−0.0263
thecut.com	−0.0262
washingtonpost.com	−0.0261
cnn.com	−0.0261
motherjones.com	−0.0259
politico.com	−0.0254
pressrun.media	−0.0253
theatlantic.com	−0.0253
courant.com	−0.0251
aclu.org	−0.0247
thebulwark.com	−0.0247
opb.org	−0.0246
argusleader.com	−0.0245
jhu.edu	−0.0245
mashable.com	−0.0240
zoom.us	−0.0239
inquirer.com	−0.0238
charlotteobserver.com	−0.0238

Table 1: The top 30 most negative PCA first component vector values and their associated domains.

Domain	First PCA Value
nypost.com	0.0723
the-sun.com	0.0715
foxnews.com	0.0712
freebeacon.com	0.0711
thefederalist.com	0.0706
twitchy.com	0.0701
dailywire.com	0.0699
townhall.com	0.0695
lawenforcementtoday.com	0.0693
washingtontimes.com	0.0692
oann.com	0.0692
trendingpolitics.com	0.0688
davidharrisjr.com	0.0686
whitehouse.gov	0.0680
americanmind.org	0.0679
foxbusiness.com	0.0679
nationalfile.com	0.0679
fxn.ws	0.0679
ussanews.com	0.0675
defendyourballot.com	0.0671
judicialwatch.org	0.0670
bongino.com	0.0669
djhjmedia.com	0.0669
washingtonexaminer.com	0.0667
breitbart.com	0.0661
par.pw	0.0660
zerohedge.com	0.0659
nationalreview.com	0.0658
Antifa.com	0.0658
donaldjtrump.com	0.0656

Table 2: The top 30 most positive PCA first component vector values and their associated domains.

1.1.2 Hierarchical clustering

Below we present the sentinel communities along the linked domain score axis colored by their clustering in the main text along with the dendrogram that resulted from hierarchical clustering.

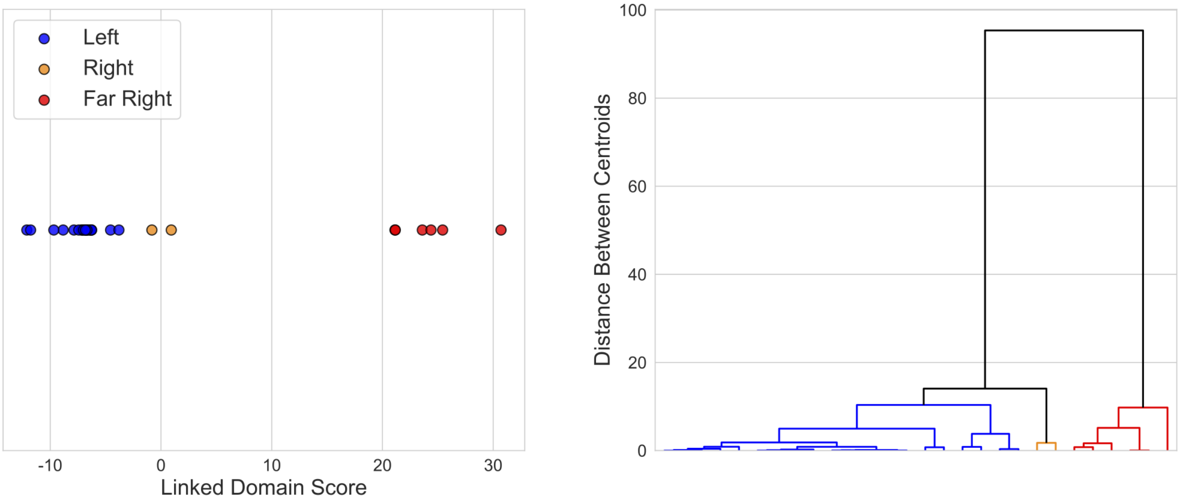

Figure 1: The linked domain score for each sentinel community colored by cluster assignment (left) together with the dendrogram produced by mean linkage clustering (right). Colors correspond to cluster assignment (Left / Right / Far Right) as described in the main text.

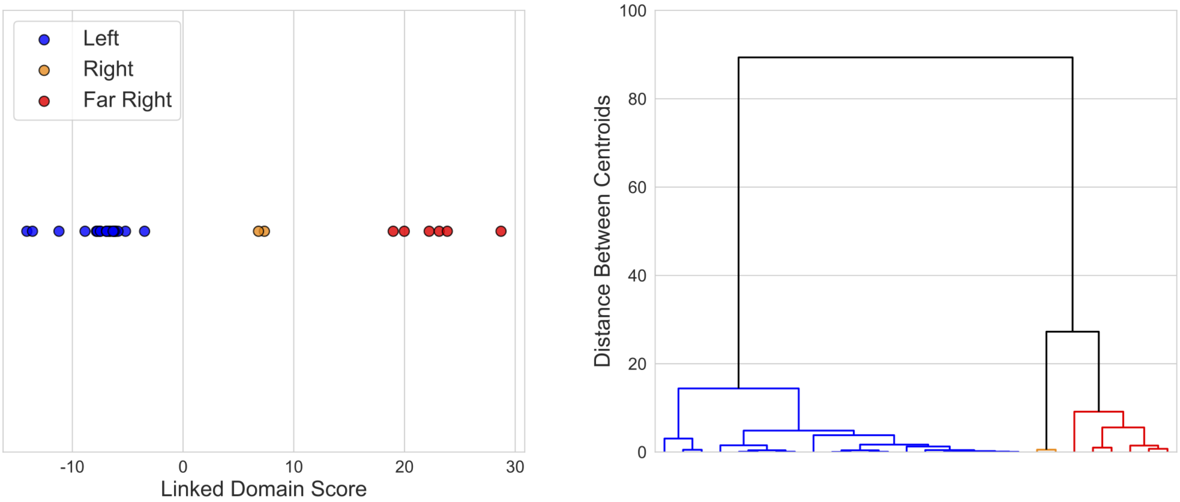

Figure 2: The linked domain score for each sentinel community (left) together with the dendrogram produced by mean linkage clustering (right), following removal of two domains that were disproportionately shared by a single sentinel node each. Colors correspond to cluster assignment (Left / Right / Far Right) as described in the main text.

The clustering we chose produced the second highest silhouette score of all possible clusterings that could result from the dendrogram in Figure 1 (0.737 in comparison to 0.894

for two clusters). This decision was made after noticing that the positioning of the two 'Right' communities was skewed left due to their disproportionate sharing of a domain connected to a single sentinel node (we check robustness of the clustering we chose to such link sharing behavior in section 1.1.3). Removing those two domains from consideration and repeating the PCA and clustering process results in the linked domain score and dendrogram seen in Figure 2.

The cut point in the dendrogram seen in Figure 2 producing three clusters has the largest silhouette score of all possible clusterings (0.814). Thus we selected a cut in the original dendrogram (Figure 1) that resulted in three clusters even though it did not correspond to the highest silhouette score.

1.1.3 Clustering robustness

To examine robustness of clustering results to linked domains stemming from a small number of nodes (for example, corresponding to self-promoting posts from a handful of accounts), we eliminated domains that were linked to by only a fraction of individual sentinels less than a given threshold. Threshold values varied from 0.0 to 0.10 in intervals of 0.005 (a stepsize of which corresponds to slightly more than two unique sentinels). For each value we repeated the PCA clustering process, and calculated an adjusted Rand index [1] comparing the resulting clusters with the Left - Right - Far Right grouping we used in our analysis. In particular, after running the thresholded domain frequency matrix through PCA we performed clustering by finding a dendrogram cut point that yielded three clusters. In Figure 3, we present the adjusted Rand index as a function of the threshold value.

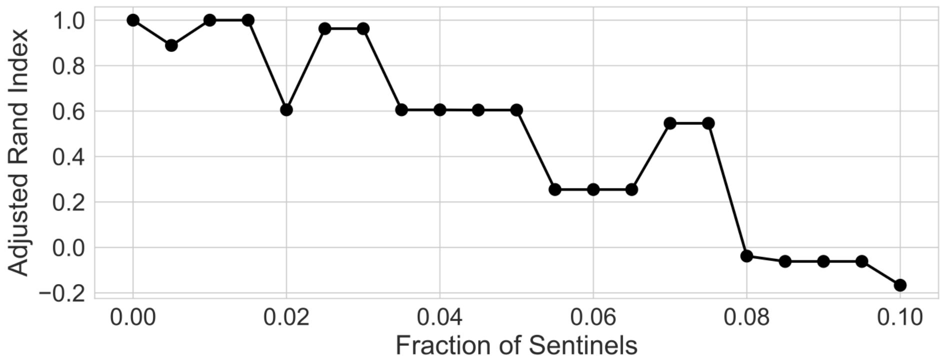

Figure 3: The adjusted Rand index comparing the clusters formed after applying PCA to the domain frequency matrix restricted to those domains posted by at least the given fraction of sentinel nodes.

The adjusted Rand index calculates the similarity of two data clusterings after adjusting for randomness in group assignment. The index ranges from -1 (completely dissimilar) to 1 (identical clusterings). As demonstrated in Figure 3 the adjusted Rand index stays at or near 1 for threshold values up to 0.03 (which represents at least 12 unique sentinels). This suggests that the clustering used in our analysis is not driven by linked domains corresponding to a handful of accounts.

1.2 Additional Sentinel Analyses

1.2.1 Sentinel coverage of known COVID-19 misinformation

By using the sentinel monitoring approach we have described we will not have captured every piece of COVID-19 misinformation circulating on the platform. However, we believe that this approach did allow for the detection of the most notable misinformation, that which reached prominent Twitter users within a diverse array of digital social circles.

We performed a series of substring searches of our sentinel tweets in order to examine topical coverage of our sentinel monitoring approach. Specifically, we examined extent of coverage by our sentinel nodes concerning virus origins, COVID-19 treatments and preventatives, three COVID-19 conspiracy theories, and non-pharmaceutical interventions (NPIs). The number of tweets returned from each cluster for every topical substring search is presented in Table 3. Search strings that produced these counts were variations on the given sub-topic name. Note that we do not include topics that were extensively examined in the main text, namely vaccinations and perceived COVID-19 severity.

Topic	Sub-Topic	Left	Right	Far Right
Virus Origins	Created in a Lab [2,3]	99	6	77
	Created by China [2,3]	21	32	68
	Bioweapon [2,3]	21	5	70
	Bat Soup [3]	34	17	3
COVID Treatments	Garlic [2]	10	0	0
& Preventatives	Vinegar [2]	1	0	1
	Vitamin C [2]	3	2	13
	Vitamin D [4]	85	27	117
	UV Light [3]	42	2	18
	Bleach [3]	269	3	56
	Hydroxychloroquine [3,5]	694	698	2,581
	Ivermectin [5]	17	5	210
	Remdesivir [5]	406	53	128
	Plasma [6]	500	67	168
Conspiracy Theories	Plandemic [3]	75	84	674
	5G [7]	40	1	23
	Population Control [3]	25	0	64
	Pandemic is Fake [2,3]	1,009	124	863
NPIs	Face Masks	1,305	189	470
	Social Distancing	11,565	1,151	4,104
	Hand Washing	338	9	50

Table 3: Number of tweets posted by each cluster that contain strings related to each subtopic. Search strings were formed by taking variations on the sub-topic labeling, e.g. "Created in a Lab" counts were found with search strings "government lab", "laboratory", "made in a lab" and "man-made".

1.2.2 QAnon posts

To quantify association of clusters with the QAnon conspiracy theory [8], we performed a substring search of all tweets posted by sentinel nodes over the observation period for those that contained at least one of the following strings: "qanon", "qarmy", "wwg", "wga", "greatawakening", "great awakening", and "new world order". These strings have been found to be affiliated with QAnon posts across various social media platforms [8, 9].

In Figure 4 we present a plot of the total QAnon tweets by sentinel account for each cluster. While all three clusters contain at least one account that posted more than 200 tweets featuring one of the sub-strings, the Far Right has many such accounts as well as a handful of users near or exceeding 1,000 found tweets.

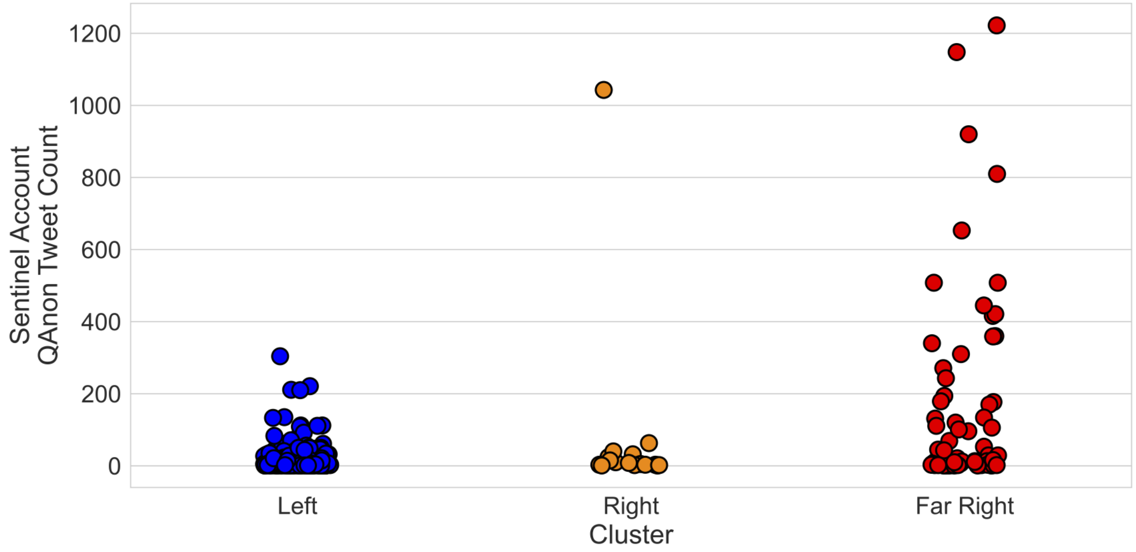

Figure 4: Number of posts containing at least one of our QAnon search strings for each sentinel distinguished by cluster. Each circle represents a unique sentinel account.

1.2.3 Sentinel drop-off

An important question for a longitudinal cohort study is the effect of subject drop-off over the observation period. In our study, drop-off may have occurred due to a sentinel leaving Twitter of their own volition or being suspended by Twitter for violating their terms of service. Over our observation period Twitter took on suspension policies that actively pursued those posting COVID-19 misinformation [10] as well as accounts determined to have been affiliated with the QAnon conspiracy theory [11]. Twitter additionally removed over 70,000 accounts on January 7, 2021 related to the storming of the United States' Capitol Building [12]. Given our analysis, these policies may explain why we observed differential attrition between the Left, Right and Far Right clusters as demonstrated in Figure 5. Attrition in this setting can be problematic because the accounts that drop off may disproportionately come from the clusters most likely to post misinformation.

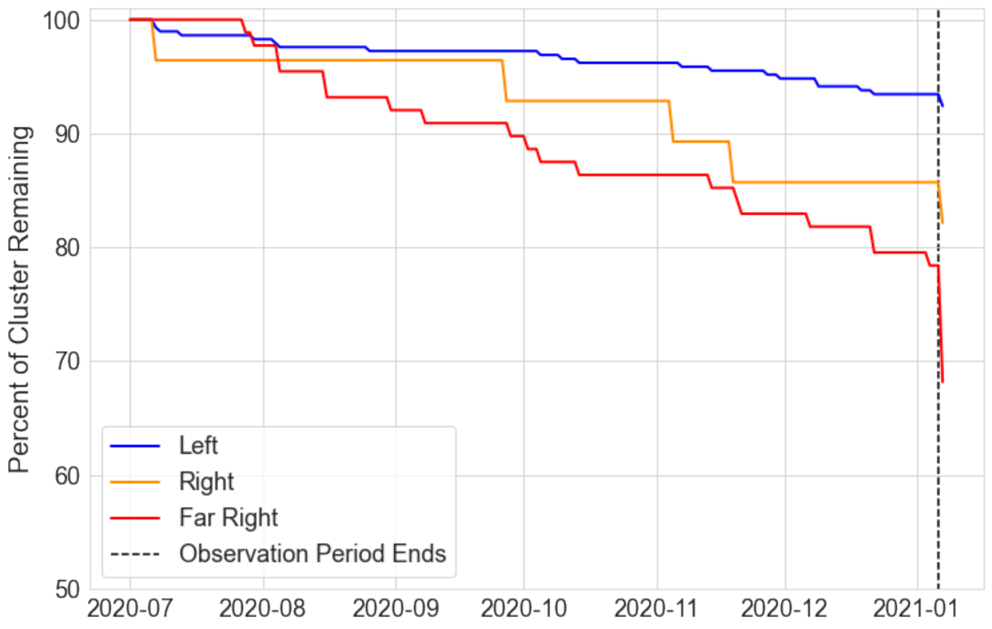

Figure 5: Sentinel attrition over the observation period by cluster. We consider a sentinel account to have been lost to follow up at the time of their last tweet within our data set.

Figure 5 shows that all clusters retained more than 80% of their initial members for all but a handful of days over our observation period, suggesting that our comparisons between clusters over time are unlikely to be skewed due to dropoff.

1.3 Search phrase tables

Below we present the search strings used to subset the COVID-19 tweets for our analysis of vaccination and perceived COVID severity content.

Table 4: Search strings used to identify COVID-19 tweets pertaining to COVID-19 severity, arranged in alphabetical order.

Strings
"case spike", "case surge", "cases spike", "confirmed cases", "coronavirus numbers", "coronavirus test", "covid count", "covid numbers", "covid spike", "covid-19 numbers", "covid-19 test", "covid19 numbers", "covid19 test", "death count", "death number", "death rate", "deaths", "die from covid", "die of covid", "die with covid", "fatality rate", "infection rate", "numbers of infected", "people infected", "positive case", "positive rate", "second wave", "spike in cases", "spikes in covid", "survival rate", "survive", "tally"

Table 5: Search strings used to subset the COVID-19 severity tweets for various sub-topics related to downplaying severity, arranged in alphabetical order.

Topic	Strings
COVID-19 is Not so Bad for the Individual	"0.1%", "94%", "99.6%", "99.9%", "a bad flu", "appears worse", "average age of death", "below normal", "below seasonal flu", "better chance of dying", "car crashes", "chances of death are super small", "children are immune", "declining in virulence", "drowning", "fear monger", "few excess deaths", "flu mortality", "from flu", "getting better quicker", "hysteria", "influenza deadlier", "isn't as deadly", "isnt as deadly", "kids are immune", "least dangerous", "less lethal", "a cold", "low mortality rate", "lower than flu", "manageable", "mild", "more dangerous than covid", "more deadly than covid", "not as contagious", "not for young", "not lethal", "nothing to fear", "obese", "obesity", "recovery rate", "seasonal flu mortality", "survival rate", "survive just fine", "than the flu", "very low risk", "we will survive", "worse than the virus"

Continued on next page

Topic	Strings
Cases and Deaths are Overreported	"6%", "100% cases positive", "9.4%", "actual cause", "additional condition", "audit", "avg age of covid death", "because of test", "being juiced', "bogus", "bullet hole", "casedemic", "cdc fraud", "chronic respiratory", "common cold", "comorbid", "coronavirus alone", "count as corona", "count as covid", "counted as a covid death", "counted as coronavirus", "counted as covid", "counted flu", "counted pneumonia", "counting a death as covid", "counting flu", "counting pneumonia", "covid alone", "covid-19 alone", "covid19 alone", "data is corrupt", "death hoax", "death scam", "deaths bs", "diagnosed with disease", "did not die from covid", "didn't die from covid", "didnt die from covid", "distort", "exaggerat", "facts don't matter", "facts dont matter", "fake test", "faking test result", "false covid death count", "false flag", "false positive", "falseflag", "faulty", "flawed estimate", "flu deaths", "flu deaths are way down", "flu is down", "flu plummet", "fraudulent", "garbage data", "gun shot", "gunshot", "heart attack", "heart disease", "inaccurate", "incorrect", "inflate", "insignificant amount", "it was the flu", "junk data", "lie on death", "lied to", "massage data", "massaging data", "massaging the data", "miscommunication", "mislead", "misreport", "mixing up coronavirus testing data", "more testing", "motorcycle", " 'new cases' ", "no second wave', "no spike", "no surge", "no testing", "non-covid causes", "not as high", "not on the rise", "not really covid", "nothing to do with #covid", "nothing to do with covid',"nursing home pandemic", "only cause of death", "other causes", "other condition", "other than covid", "other than the coronavirus', "overcount", "padded", "padding", "pneumonia", "poisoning", "preexisting condition", "pre-positive", "preexisting condition", "quietly update", "real cause of death", "real covid death numbers", "real infection rate', "real number", "reclass", "second wave fake", "secondary conditions", "spike in test", "suspect", "suspicious", "testing is so massive", "tests don't work", "tests dont work", "the facts", "trash data", "under 0.2%", "underlying conditions", "unintentional injury', "unrelated to covid", "useless test", "weren't covid", "werent covid", "with a sniffle", "with the virus", "wont believe any of the data"
The Pandemic is a Hoax or Fabricated	"covid is over", "doesn't exist", "doesnt exist", "empty hospital", "expose their lies", "false alarm", "hoax", "magically goes away", "no pandemic", "pandemic is fake",' 'pandemic is over", "simulation"

Table 6: Search strings used to identify COVID-19 tweets pertaining to vaccinations, arranged in alphabetical order.

Strings
"astrazeneca", "biontech", "johnson & johnson", "johnson and johnson", "moderna", "pfizer", "vaccinat", "vaccine"

Table 7: Search strings used to identify tweets mentioning vaccine misinformation or related to vaccine hesitancy, arranged in alphabetical order.

Topic	Terms/Phrases
Vaccine Misinformation	"a tracker", "abort", "ai software", "alter dna", "babies", "baby", "bio terrorist", "bioterrorist", "bioweapon", "brainwash", "cabal", "cause hiv", "causes hiv", "change dna", "change human dna", "chip", "coronascam", "corrupt dna", "covid was planned", "covidscam", "crime against humanity", "crimes against humanity", "deep state", "depopulat", "disable", "dna alter", "dna chang", "dna corrupt", "dna modif", "dna mutate", "dna transform", "eugenic", "fertil", "fetal", "fetus", "flu vaccination and covid", "foreign mrna", "franken vax","frankenstein vax", "gene therapy", "genetically modif", "genocid", "guinea pig", "hiv in it", "hoax", "insect cell", "low population", "lower population", "lower the population", "mark of the beast", "microchip", "mikovits", "modified dna", "modify dna", "monkey cell", "monkey dna", "mutate dna", "mutate your dna", "my dna", "nanoparticle", "nano-tube", "nanoparticle", "nanotube", "pandemrix", "placenta", "plandemic", "population control", "population low", "population reduc", "reduce population", "reduce the population", "satellite surveilance", "scam", "sensor", "sham ", "special ai", "steril", "tracer", "trackers", "transform dna", "transhuman", "two-way", "unborn"
Vaccine Hesitancy	"adverse", "aluminum", "avoid", "bells palsy", "bobby kennedy", "brain damage", "danger", "defective", "dont tak", "experimental", "facial paralysis", "formaldehyde", "hard pass", "harm", "i refuse", "i will refuse", "i'm not", "im not", "maim", "mercury", "multiple sclerosis", "nightmar", "no need for", "no thank", "not tak", "nurse faint", "pass on", "poison", "rfk", "robert f kennedy", "robert f. kennedy", "robert kennedy", "rushed", "rushing", "say no", "sinister", "spinal chord", "spinal cord", "stay away", "tachycardia", "toxic", "transverse myelitis", "unproven", "unsafe", "vaccine is dangerous", "will not take", "won't take", "wont take", "would not take", "wouldn't take", "wouldnt take"

1.4 Additional flagged day material

1.4.1 Augmented Dickey -Fuller test results

The burst score metric in equation (1) of the main text implicitly assumes that the similarity between two clusters is stationary in time and does not exhibit a trend. In order to assess the validity of this assumption we performed an augmented Dickey-Fuller test [13] with no trend and no lag using the `adfuller` model in the statsmodels Python package [14]. For the Right - Far Right between similarity this test resulted in a test statistic value of -11.165 with a corresponding p-value of 2.722×10^{-20}, indicating that there is significant evidence to reject the null hypothesis that the time series has a unit root in favor of the alternative that the time series is stationary.

1.4.2 Flagged day table

In the main text we provided a figure with all of the flagged days over the observation period. We considered a flagged day to be any day in which the Far Right and Right clusters exhibited a burst score of at least two. For each flagged day we gave a short description of the "topic" driving increased similarity on that day. Here we elaborate further on each flagged day with the date, a longer description of the topic on that day as well as the original Right - Far Right between similarity score on that day followed by a recalculated score found by calculating the average between similarity after removing the topical tweets (as determined by latent semantic analysis). This information is provided in Table 8.

**Date**	**Topic**	**Original Score**	**Removed Score**
8/3/2020	Nevada governor using COVID to "steal the election"	0.093	0.022
8/30/2020	CDC "quietly" updating the death statistics	0.19	0.055
10/2/2020	Donald Trump announces COVID diagnosis	0.15	0.031
10/5/2020	Donald Trump leaves Walter Reed Medical Center and Press Secretary Kayleigh McEnany tests positive for COVID-19	0.10	0.040
11/7/2020	Michigan governor has poll watchers removed	0.12	0.024
11/13/2020	Elon Musk calls COVID tests into question	0.11	0.040
11/22/2020	Maryland governor purchased faulty COVID tests	0.090	0.019
12/21/2020	COVID relief bill passed and child sex trafficking discussion	0.091	0.026
1/3/2021	Donald Trump claims CDC is exaggerating COVID deaths	0.11	0.032

Table 8: Descriptions of the flagged days from Figure 6, main text. Topics were determined by running the tweets from each cluster sent on that date through latent semantic analysis and reading the tweets with highest singular vector values. The original score refers to the Right - Far Right inter-similarity score on that day, and the removed score is the recalculated inter-similarity score after topical tweets were removed.

The topics driving increased similarity tend to fall into two categories: topics related to political discourse involving COVID-19, and topics downplaying COVID-19 severity.

1.4.3 Flagged day analysis

November 13, 2020 - Elon Musk tweets about His COVID tests High between cluster similarity on November 13, 2020 was driven by content related to a tweet sent by Tesla CEO Elon Musk on that day that harshly criticized the reliability of rapid antigen tests. The observed similarity between the Right and Far Right on this day was 0.11, roughly 3 standard deviations from the average similarity up to that time. In Figure 6 we plot the cumulative per community tweets containing Elon Musk's name along with a vertical dashed line denoting the time of Mr. Musk's original tweet.

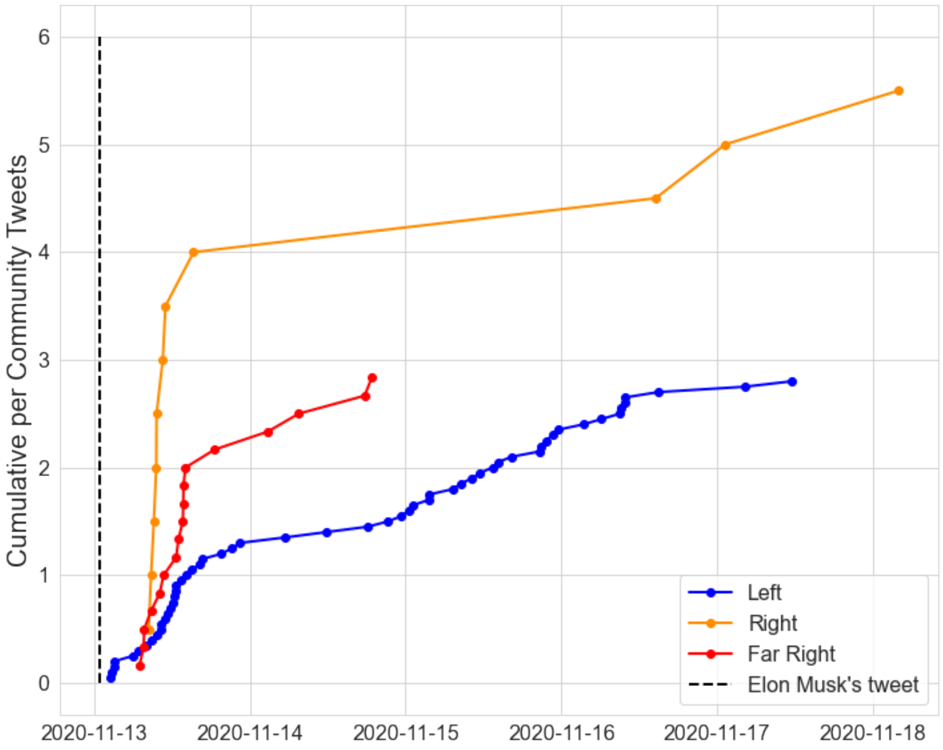

Figure 6: Cumulative per community tweet curves for each cluster for tweets about Elon Musk's questioning of COVID rapid antigen tests. The vertical black dotted line denotes the time of his tweet.

This topic sees roughly equal volume across all three clusters, with close to identical timing. However, the nature of the engagement differs by cluster. A number of sentinel accounts in the Right and Far Right clusters endorsed his tweet while calling the tests, and COVID in general, a scam. Conversely the Left's tweets consist of news reports of Mr. Musk's positive test and criticism of his downplaying the virus.

January 3, 2021 - "exaggerated" COVID-19 deaths Right - Far Right similarity on this day was observed to be 0.11, roughly 3 standard deviations above the historic score. The

topic driving increased Right - Far Right inter-similarity on January 3, 2021 was reaction to a tweet by Donald Trump which claimed that the number of cases and deaths reported by the Centers for Disease Control were exaggerated. We present a cumulative per community tweet plot for tweets about this topic in Figure 7.

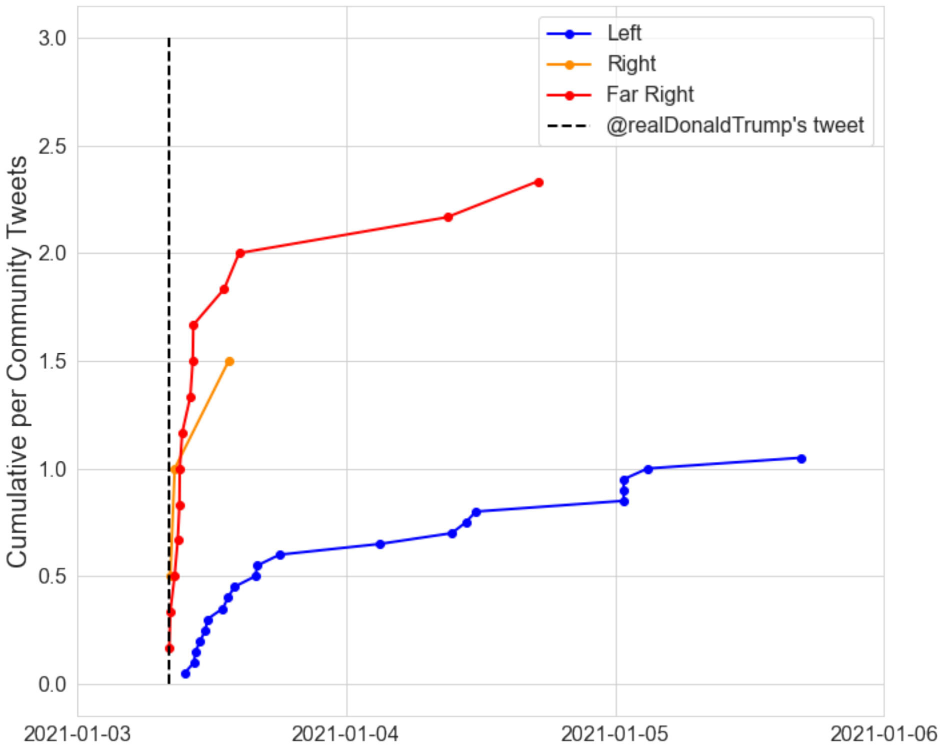

Figure 7: Cumulative per community tweet curves for each cluster for tweets reacting to Donald Trump's exaggerated counts tweet. The vertical black dotted line denotes when the President Trump posted his tweet.

Examination of tweets from the Right and Far Right reveals a number of straight retweets of President Trump's original tweet as well as original sentinel tweets in agreement with the sentiment. Meanwhile the Left's tweets largely consist of accounts stating the danger of Donald Trump's tweet in addition to reports of Dr. Fauci and the United States Surgeon General contradicting his claim.

References

[1] L. Hubert and P. Arabie, "Comparing partitions," Journal of Classification, vol. 2, no. 1, pp. 193–218, 1985.

[2] G. Pennycook, J. McPhetres, B. Bago, and D. G. Rand, "Beliefs about COVID-19 in Canada, the United Kingdom, and the United States: A novel test of political polarization and motivated reasoning," Personality and Social Psychology Bulletin, p. 01461672211023652, 2021. PMID: 34180276.

[3] S. Evanega, M. Lynas, J. Adams, K. Smolenyak, and Cision Global Insights, "Coronavirus misinformation: quantifying sources and themes in the COVID-19 'infodemic'," JMIR Preprints, 2020.

[4] J. Henrina, M. A. Lim, and R. Pranata, "COVID-19 and misinformation: how an infodemic fuelled the prominence of vitamin D," British Journal of Nutrition, vol. 125, no. 3, pp. 359–360, 2021.

[5] D. A. Erku, S. A. Belachew, S. Abrha, M. Sinnollareddy, J. Thomas, K. J. Steadman, and W. H. Tesfaye, "When fear and misinformation go viral: pharmacists' role in deterring medication misinformation during the 'infodemic' surrounding COVID-19," Research in Social and Administrative Pharmacy, vol. 17, no. 1, pp. 1954–1963, 2021.

[6] P. Rzymski, L. Borkowski, M. Drag, R. Flisiak, J. Jemielity, J. Krajewski, A. Mastalerz-Migas, A. Matyja, K. Pyrć, K. Simon, M. Sutkowski, J. Wysocki, J. Zajkowska, and A. Fal, "The strategies to support the COVID-19 vaccination with evidence-based communication and tackling misinformation," Vaccines, vol. 9, no. 2, p. 109, 2021.

[7] A. M. Enders, J. E. Uscinski, C. Klofstad, and J. Stoler, "The different forms of COVID-19 misinformation and their consequences," The Harvard Kennedy School Misinformation Review, vol. 1, no. 8, 2020.

[8] M. Aliapoulios, A. Papasavva, C. Ballard, E. De Cristofaro, G. Stringhini, S. Zannettou, and J. Blackburn, "The gospel according to Q: Understanding the QAnon conspiracy from the perspective of canonical information," arXiv:2101.08750, 2021.

[9] A. Papasavva, J. Blackburn, G. Stringhini, S. Zannettou, and E. De Cristofaro, ""Is it a Qoincidence?": A First Step Towards Understanding and Characterizing the QAnon Movement on Voat.co," arXiv:2009.04885, 2020.

[10] "COVID-19 misleading information policy." https://help.twitter.com/en/rules-and-policies/medical-misinformation-policy, 2020.

[11] K. Conger, "Twitter Takedown Targets QAnon Accounts." https://www.nytimes.com/2020/07/21/technology/twitter-bans-qanon-accounts.html, 2020.

[12] T. Romm and E. Dwoskin, "Twitter purged more than 70,000 accounts affiliated with QAnon following Capitol riot." https://www.washingtonpost.com/technology/2021/01/11/trump-twitter-ban/, 2021.

[13] D. A. Dickey and W. A. Fuller, "Distribution of the estimators for autoregressive time series with a unit root," Journal of the American Statistical Association, vol. 74, no. 366a, pp. 427–431, 1979.

[14] S. Seabold and J. Perktold, "statsmodels: Econometric and statistical modeling with Python," in 9th Python in Science Conference, 2010.

Instructions for Undergraduate Coders

Introduction

In this file are instructions for undergraduate coders. Within we will describe the data, what job the undergraduate coders will perform, and provide guidance as to how they should accomplish that job.

The Data Set

The data you will be coding contains 800 tweets spanning four distinct topics (200 tweets per topic) regarding COVID-19. You will be presented (in a Qualtrics survey) the tweet as well as the topic associated with the tweet. *Note that it is possible for you to see the same tweet appear with different topics.*

The four topics include:

1. Plandemic
2. Hydroxychloroquine
3. Mask Wearing
4. COVID-19 mortality

Coding Online

Each coder will have their own online survey link they will use to code each tweet.

The steps are:

1. Click on the link
2. Paste in tweet id # and tweet text from the excel sheet
3. Answer the questions about the tweet content as noted on the survey and coding sheet below
4. Hit submit
5. Re-open link and start again with the next tweet
6. Complete process for all 800 tweets

7. Keep track of progress in completing the tweets on the excel sheet so you do not code more than once

Coding

For each tweet you view you will be asked to answer the following four questions:

- Does this tweet present misinformation? (*Yes / No / Unsure*)
    - *We define misinformation as false or inaccurate information in accordance with the facts we present below.*
- Does this tweet reference a statistic? (*Yes / No*)
    - *Example: The average American generates nearly 4.5 pounds of trash each day.*
    - *Example: There are roughly half a million pieces of space junk in orbit around the Earth that measure at least half an inch wide.*
- Does this tweet downplay/dampen or play up/amplify the health risks associated with COVID-19 infection? (*Amplify Severity/ Downplay Severity / Neutral/ Unknown or Does not mention*)
- Is this tweet about the topic it is associated with? (*Yes / No/Unsure*)
- Does this tweet include a “Call to Action”. This is defined as mobilizing information, either (A) calling for in person collective action (protest, march, sit-in, etc), or (B) online collective action (signing online petition, asking people to share or reposting a video, meme, article, etc.) or (C) sharing information about how to take collection action (e.g. a link, contact information of an official/time/place of a protest etc.) (Yes/No and code type of mobilizing information (in person, online, sharing information)
- For tweets related to hydroxychloroquine and mask wearing you will also be asked to respond to the following: Does this tweet downplay or play up the effectiveness of hydroxychloroquine (or facemasks)? (*More effective / Less effective / Neutral*)
- For tweets about mortality rate, you will be asked: Does this tweet make claims that the mortality rate is lower, higher, or neutral than health experts suggest? (Mortality Rate is Lower / Mortality Rate is Higher / Neutral / Unsure)

Note that we define what we mean by misinformation below.

Coding Instructions

In this section we will provide context and give a list of the misinformation you will likely see along with the facts corresponding to said misinformation. Again *we define misinformation as false or inaccurate information in accordance with the facts we present below.*

We will first present a few pieces of misinformation that you may encounter across all four topics. We will then dive into topic-specific misinformation. For each of the four topics we will provide a brief background, references, and a list of common pieces of misinformation that you may encounter.

General Misinformation

In this section we will briefly describe some general misinformation that could appear in any of the four topics. For each item below the misinformation will be presented and then followed by the truth.

Misinformation	Truth
The pandemic was planned	There is no evidence of this
The pandemic is fake or a hoax	The COVID-19 pandemic is a real pandemic
Hydroxychloroquine is a COVID cure	The scientific consensus is that hydroxychloroquine is not effective for treating or preventing COVID-19

Plandemic

<u>Summary of Plandemic</u>

The Plandemic Video - Plandemic was a widely-viewed video posted in May of 2020 in which obscure filmmaker Mikki Willis interviewed Dr. Judy Mikovits, a former scientist at the National Cancer Institute. A longer version of the documentary (Pandemic: Indoctornation) was released on August 18, 2020.

Dr. Mikovits - Dr. Mikovits gained notoriety in the late 2000s for a study published in Science linking Chronic Fatigue Syndrome to xenotropic murine leukemia virus-related virus. This study was later discredited and Science retracted the article. However, Dr. Mikovits refused to sign the retraction notice. In 2011 Dr. Mikovits' former employer filed a suit against her and she was eventually arrested on felony charges in California. Criminal charges were eventually dropped against her. In July 2020 Sinclair

Broadcasting Group (SBG) scheduled an interview with Dr. Mikovits. In this interview Mikovits asserted that Fauci created COVID-19.

Common Misinformation

Misinformation	Truth
Health and Human Services colluded with the Department of Justice and FBI to destroy Dr. Mikovits' reputation.	There is no evidence of this.
Health and Human Services colluded with the Department of Justice and FBI to destroy Dr. Mikovits' reputation.	There is no evidence of this.
Dr. Anthony Fauci directed aforementioned efforts.	There is no evidence of this.
Dr. Fauci delayed previous publications of Dr. Mikovits on HIV which benefitted Fauci and his friends while leading to the death of millions.	There is no evidence of this.
COVID-19 was manipulated in a lab and released into the world, either by accident or as a bioweapon.	Scientific consensus supports the theory that COVID-19 jumped from animals into human hosts in the wild.
Hospitals make money from Medicare if they label a death as being due to COVID-19.	Medicare does give money to hospitals that treat coronavirus patients. However, this is a standard practice for all diseases, and there is no indication that hospitals are over-identifying patients as having COVID-19 in an attempt to make money.
Getting a flu shot increases the odds that you'll contract COVID-19.	There is no evidence of this.
Hydroxychloroquine is an effective treatment against coronaviruses.	Evidence from various scientific studies suggests that hydroxychloroquine is no more effective than any other treatments that have been considered.

Dr. Anthony Fauci created the virus and sent it to China.	This is no evidence of this.

References
https://www.sciencemag.org/news/2020/05/fact-checking-judy-mikovits-controversial-virologist-attacking-anthony-fauci-viral
https://www.politifact.com/article/2020/may/08/fact-checking-plandemic-documentary-full-false-con/
https://apnews.com/article/ap-top-news-eric-bolling-anthony-fauci-entertainment-politics-d49a45e68eebaf5f021b685142530819

Hydroxychloroquine

Summary of Hydroxychloroquine

In March 2020 Dr. Didier Raoult announced a study in which he claimed the use of hydroxychloroquine and azithromycin was effective in treating COVID-19. His study was found "irresponsible" by peer reviewers. Shortly after Raoult's announcement President Trump started promoting hydroxychloroquine as a COVID treatment. This led to a surge in off-label prescriptions of the drug. On March 28, 2020 the FDA issued an emergency use authorization to allow hydroxychloroquine to be prescribed to patients hospitalized with COVID-19. In May of 2020 President Trump stated he was taking hydroxychloroquine combined with zinc and an initial dose of azithromycin. On May 22, 2020 a study published in the Lancet raised concerns on the safety of prescribing hydroxychloroquine to treat COVID-19. The Lancet study was later retracted in June. On June 15 the FDA revoked emergency use authorization. June also marked a period in which more large-scale studies began to suggest that hydroxychloroquine was not an effective treatment for COVID-19.

Common Misinformation on Hydroxychloroquine

Misinformation	Truth
Hydroxychloroquine cures COVID-19.	There is no scientific evidence that hydroxychloroquine cures COVID-19. Also at the time of this writing there is no scientifically proven cure for COVID-19.
Hydroxychloroquine prevents COVID-19.	There is no scientific evidence that hydroxychloroquine prevents COVID-19. Also at the time of this writing there is no

	scientifically proven prevention for COVID-19, although several vaccines are in phase three clinical trials.
Hydroxychloroquine/chloroquine is safe.	According to the FDA there are reports of serious heart rhythm problems and other safety issues, including blood and lymph system disorders, kidney injuries, and liver problems and failure associated with hydroxychloroquine treatment for hospitalized COVID-19. patients. Further, there may be mild side effects including nausea, occasional vomiting, or diarrhea. Prolonged use may also result in eye damage.
Pharmaceutical companies, Bill Gates, Anthony Fauci, and various others don't want hydroxychloroquine to be prescribed because of potential financial gain associated with other treatments	There is no evidence of this.

References

https://abcnews.go.com/Health/timeline-tracking-trump-alongside-scientific-developments-hydroxychloroquine/story?id=72170553
https://en.wikipedia.org/wiki/Hydroxychloroquine#COVID-19
https://www.sciencemag.org/news/2020/06/three-big-studies-dim-hopes-hydroxychloroquine-can-treat-or-prevent-covid-19
https://www.fda.gov/drugs/drug-safety-and-availability/fda-cautions-against-use-hydroxychloroquine-or-chloroquine-covid-19-outside-hospital-setting-or
https://www.cdc.gov/malaria/resources/pdf/fsp/drugs/Hydroxychloroquine.pdf?fbclid=IwAR1lVp5ucnLK9g_crh1lro-BHlgFiqXT7sOP30lNVtTLvWCxZT5dcbB4MuM

Masks

Summary of Mask Wearing Recommendations During COVID19

CDC - Prior to April 3, 2020 the CDC did not recommend the wearing of face masks to prevent the transmission of COVID19. As of April 3, 2020 the CDC updated their recommendations to say that people should wear a cloth face covering in public. This was further updated on June 28, 2020 to recommend that people wear cloth face coverings in public settings and when around people who don't live in their household,

especially when other social distancing measures are difficult to maintain. It is the position of the CDC (which is backed up by various scientific studies) that widespread proper use of facemarks is likely to reduce the spread of COVID-19 in public settings.

The World Health Organization (WHO) - Prior to June 5, 2020 WHO did not actively recommend that people wear facemasks in public. On June 5, 2020 WHO guidance was updated to recommend that the general public should wear non-medical fabric masks where social distancing is not possible and vulnerable people should wear medical masks in such settings.

Efficacy of Masks - Early in the pandemic there was little to no research on the effectiveness of face masks in decreasing COVID19 transmission. In the summer of 2020 the scientific consensus became that widespread proper face mask utilization is effective in reducing COVID19 transmission.

Correct Way to Wear a Mask - In order to be effective, face masks should cover both the nose and mouth.

Common Mask Misinformation

Misinformation	Truth
Face masks are worse than doing nothing.	While the effectiveness of a particular mask depends on a number of factors (for example, an N95 mask is more effective than a cloth mask) it is the current scientific consensus that properly using a face mask is better than nothing.
Duke University Study shows Many Face Coverings INCREASE Transmission of COVID-19.	This is referring to a misinterpreted study conducted by Duke University researchers that proposed a new method for testing the effectiveness of different face coverings. This study says nothing statistically significant about the effectiveness of different coverings.
Face masks are a way for the government (or others) to infringe on individual rights and freedoms.	Face mask recommendations are made in the interest of preventing the spread of COVID-19. There is no evidence of a plot

	to control the freedoms of American citizens using face masks.
Anthony Fauci said masks don't work.	While Dr. Fauci did say not to wear face masks in a 60 minutes interview on March 8, this statement was made before the CDC altered their guidelines and before there was a better understanding of mechanisms for COVID-19 spread. Dr. Fauci has since changed his position as more evidence has illuminated how the virus spreads from person to person.
Face masks reduce oxygen intake and increase the amount of carbon dioxide you breathe in.	This is false. Masks may be uncomfortable and increase anxiety, but they do not reduce oxygen intake or increase your carbon dioxide levels.

References

https://www.bjc.org/Coronavirus/Information-Resources/ArtMID/5707/ArticleID/4430/CDC-issues-new-guidelines-on-wearing-cloth-face-masks-in-public

https://www.nature.com/articles/d41586-020-02801-8

https://en.wikipedia.org/wiki/Face_masks_during_the_COVID-19_pandemic

https://www.newsweek.com/fact-check-did-dr-fauci-say-no-masks-like-trump-claiming-1540383

https://medical.mit.edu/covid-19-updates/2020/08/neck-gaiters

https://wexnermedical.osu.edu/blog/masks-oxygen-levels

COVID-19 Mortality

Summary of COVID-19 Mortality

Starting in March/April 2020 the belief that the COVID-19 death toll was not as severe as was being reported started to circulate. In particular, believers of this theory postulated that deaths due to other causes unrelated to COVID-19, like heart attacks and gunshot wounds, were being erroneously recorded as COVID-19 deaths on official death certificates. According to the theory, this was being done in order to stoke fear within the population as a way to infringe on individual freedoms and rights.

In the summer of 2020 this belief grew as people started to misread official CDC COVID-19 data, and incorrectly claim that only 6% of the reported COVID-19 fatalities

were actually due to COVID-19. This claim comes from CDC data posted in August 2020 which stated that of all deaths attributed to COVID-19 at that time only 6% were patients that only had COVID-19. The remaining 94% also had at least one other condition which may or may not have contributed to their death.

There is also the widely-held belief that COVID-19 is no worse than seasonal influenza (flu). In particular, many claim that the flu kills more people each year than COVID-19 has during the pandemic. This has been shown to be incorrect. Examining CDC influenza death statistics for the past 11 flu seasons demonstrates that in each of those years the flu killed fewer than 100,000 Americans. In contrast the CDC reported 100,000 American COVID-19 deaths on May 27, 2020, less than six months after the first known American diagnosis of COVID-19.

For most of the pandemic the severity of COVID-19 has been downplayed by some with the common refrain that 99% or 99.9% of those that get the disease survive. In reality the COVID-19 survival rate is dependent upon a number of factors including age, body mass index, and underlying health conditions.

<u>Common Misinformation</u>

Misinformation	Truth
COVID-19 death statistics have been over reported by doctors for political and/or financial gain.	There is no evidence of systematic false reporting of COVID19 deaths by medical professionals. These claims have been refuted by numerous medical professional organizations.
The CDC quietly updated their mortality statistics to cover up the real mortality rates.	This is false, see the above summary for an explanation.
The flu kills more people annually than COVID-19 has killed.	This is false, with the caveat that early on in the pandemic covid deaths were fewer than annual flu fatalities. According to the CDC fewer than 100,000 individuals died from the flu in each of the past 11 flu seasons. In contrast COVID-19 reached this mark on May 27, 2020.

Only old people die from COVID19.	This is false. Anyone can die from COVID-19, but it is true that some demographics are statistically more likely to die from the disease.

Sources

https://www.rollingstone.com/culture/culture-features/anti-vax-doctor-covid-19-death-certificates-984407/
https://www.snopes.com/fact-check/cdc-mortality-statistics/
https://www.cdc.gov/nchs/nvss/vsrr/covid19/index.htm
https://www.nbcnews.com/think/opinion/truth-about-cdc-s-covid-19-death-rate-conspiracies-undermining-ncna1241343
https://www.reuters.com/article/us-health-coronavirus-usa-deaths/u-s-covid-19-deaths-likely-higher-than-reported-study-shows-idUSKBN2426GZ
https://jamanetwork.com/journals/jamainternalmedicine/fullarticle/2767980
https://www.medrxiv.org/content/10.1101/2020.08.31.20184036v3.full.pdf+html
https://twitter.com/choo_ek/status/1320513892208930817
https://www.cdc.gov/media/releases/2020/s0528-coronavirus-death-toll.html
https://wwwnc.cdc.gov/eid/article/26/6/20-0516_article#:~:text=A%20patient%20in%20the%20United,January%2020%2C%202020.
